# Viewpoint: A Contributory Role of Shell Ginger (*Alpinia zerumbet*) for Human Longevity in Okinawa, Japan?

**DOI:** 10.3390/nu10020166

**Published:** 2018-01-31

**Authors:** Rolf Teschke, Tran Dang Xuan

**Affiliations:** 1 Department of Internal Medicine II, Division of Gastroenterology and Hepatology, Klinikum Hanau, D-63450 Hanau, Teaching Hospital of the Medical Faculty, Goethe University Frankfurt/ Main, Frankfurt/ Main, Germany; 2 Division of Development Technology, Graduate School for International Development and Cooperation (IDEC), Hiroshima University, Higashi Hiroshima 739-8529, Japan; tdxuan@hiroshima-u.ac.jp

**Keywords:** *Alpinia zerumbet*, shell ginger, longevity, centenarians, Okinawa, Okinawa diet, bioactive phytochemicals, radical scavenging chemicals, reactive oxygen species, ROS, antioxidants, polyphenols, kavalactones, sirtuins

## Abstract

The longevity of the population in the Okinawa Islands of Japan has been ascribed to genetic factors and the traditional Okinawa cuisine, which is low in calories and high in plant content. This diet includes shell ginger (*Alpinia zerumbet* (Pers.) B.L. Burtt & R.M. Sm) of the ginger family (Zingiberaceae). Due to its local popularity, *Alpinia zerumbet* has become the subject of a good deal of study at the University of the Ryukyus in Okinawa. Personal local experience and review of the literature now suggest that culinary shell ginger may contribute to longevity among the population in Okinawa. This is supported by its abundant phytochemical content, with antioxidant and anti-obesity properties. The major bioactive phytochemicals are dihydro-5,6-dehydrokawain (DDK; 80–410 mg g^−1^ fresh weight), 5,6-dehydrokawain (DK; ≤100 mg g^−1^), and essential oils, phenols, phenolic acids, and fatty acids (≤150 mg g^−1^ each). Further, *Alpinia zerumbet* extends the lifespan in animals by 22.6%. In conclusion, culinary shell ginger may significantly contribute to human longevity in Okinawa.

## 1. Introduction

Japan has world’s highest life expectancy, at 83.4 years (females 86.6 years and males 80.1 years) according to the World Health Organization (WHO) [1,2]. Other countries in the top ten with respect to life expectancy are Spain, Switzerland, Italy, Australia, Israel, Iceland, Sweden, Luxembourg, and Norway. For almost 30 years (prior to 2000), Okinawa had the longest life expectancy of all prefectures in Japan. Since then, life expectancy of Okinawans declined due to factors that include the increasing Westernization of the lifestyle [3,4,5]. Prevalence data of centenarians in the Okinawa archipelago with its 1.3 million inhabitants are variable, but estimates from the Okinawa International University in Ginowan, Okinawa, suggest approximately 50 centenarians per 100,000 persons [6], amounting to about 650 centenarians in Okinawa. This is about 4–5 times the average for most Western countries [6]. The longevity of Okinawans has been linked to various conditions, including the local cuisine—the “Okinawa diet” [5].

In the past years, several reports have focused on Okinawan longevity and its causes [3,4,5,7,8,9]. In addition, researchers (above all from the University of the Ryukyus, Okinawa) have been interested in characterization of a particular plant, namely shell ginger (*Alpinia zerumbet* (Pers.) B.L. Burtt & R.M. Sm, in short *A. zerumbet*) [10,11,12,13,14,15,16,17,18,19]. This plant is typical in Okinawa as it is cultivated in the gardens and fields in the area, or else grows naturally throughout the Okinawan Islands, syn. Islands of the Ryukyus. Shell ginger is a plant broadly used in the traditional Okinawan cuisine and as a traditional herbal medicine. Its bioactive phytochemicals have been identified, isolated from the crude plant, synthetized, and characterized in detail [18,19]. Recent experimental studies revealed that *A. zerumbet* substantially expanded the lifespan in animals [20].

In the present article, we focus on shell ginger and its possible contributing effect towards the longevity of the population in Okinawa and discuss in detail such a potential relationship. Our analysis is based on a thorough assessment of the relevant scientific literature, and also includes our personal and professional local experience in the Okinawa Islands.

## 2. Search Strategy

### 2.1. Search and Identification Terms

The PubMed database was searched for identification of original publications and review articles referring to clinical, epidemiological, botanical, and experimental studies, using the following terms alone or combined: longevity, centenarians, Okinawa, Okinawa diet, *Alpinia zerumbet*, and shell ginger. The literature search was performed during October and November 2017.

### 2.2. Data Analysis

Publications were analysed for their clinical and scientific value, data quality, and relevance in relation to the topic of this article. Publications of good quality and in the English language were preferred and considered for evaluation, but some important studies published in other languages such as Japanese were also included in the analysis. Based on the retrieved publications and their reference lists, a manual search was added with a focus on reports not yet identified. 

## 3. *Alpinia* Species

### 3.1. The Alpinia Genus and the Zingiberaceae (Ginger) Family

*Alpinia* is the largest genus of the family Zingiberaceae (ginger), classified by Charles Plumier and named after Prospero Alpino, a well-known Italian botanist of the sixteenth century [21]. Plants of the genus *Alpinia* and their constituents have numerous positive effects, including antimicrobial, antiparasitic, insecticidal, anticancer, antiproliferative, antiinflammatory, analgesic, antiallergic, neuroprotective, and antioxidant properties, as summarized previously [21]. Some plants of the *Alpinia* genus also may exert specific beneficial effects. Under discussion are abundant diseases or health conditions, leading to the proposal that *Alpinia* may even be a goldmine for future therapies [21]. As one of the most important genera of the Zingiberaceae family with around 230 species, *Alpinia* had received attention in 252 publications by 2015, with 544 compounds isolated overall from 35 *Alpinia* species, as described in detail [22].

### 3.2. Alpinia Zerumbet as A Plant

**Figure 1:**
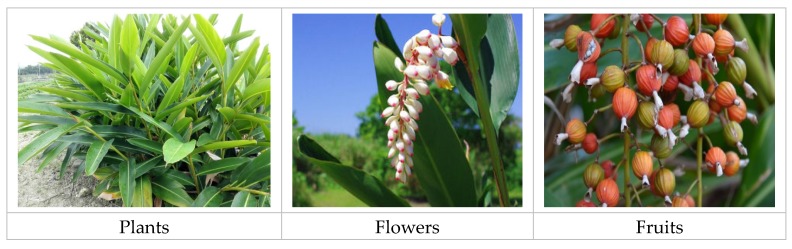
Photos of the plant *Alpinia zerumbet* grown in Okinawa.

The plant *A. zerumbet* (Pers.) B.L. Burtt & R.M. Sm, syn. *Alpinia speciosa,* also called shell ginger, belongs to the large *Alpinia* genus. It grows widely in tropical and subtropical regions including the Okinawa Islands [14,15,16,17,18,19,20], where *A. zerumbet* can easily be studied, allowing for a detailed botanical description. With a height of up to 3.0 m in the free landscape, *A. zerumbet* is a perennial herb with rhizomes, which are aromatic when cut, with a typical ginger smell. The leaves are two-ranked and thin, approximately 25–35 cm long. Appreciated also as indoor plant with a smaller height, the flowers of this beautiful plant are pink when in bud, and resemble seashells, which thus led to the synonyms shell ginger, shell flower, pink porcelain lily, variegated ginger, butterfly ginger, and light galangal. The fruits are ball-shaped with striations of initially yellow colour, changing to red as the fruit matures (Figure 1).

Presently, in common use are the leaves and rhizomes of *A. zerumbet* for traditional Okinawa cuisine and herbal medicine. Based on current literature, all parts seem to be edible, but data on maximum amounts tolerated by humans without signs of toxicity are not available. The histogram of *Alpinia zerumbet* is presented in Table 1.

## 4. Phytochemicals of *Alpinia Zerumbet*

**Figure 2:**
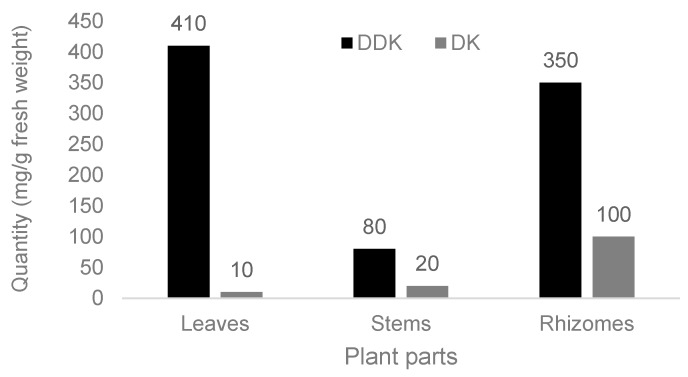
Amounts of dihydro-5,6-dehydrokavain (DDK) and 5,6-dehydrokawain (DK) in different plant parts of *Alpinia zerumbet*, modified from the data of Tawata et al. [18].

*A. zerumbet* is rich in bioactive phytochemicals, with complex structures belonging to two groups, the kavalactones including two major kavalactones (Figure 2) and the non-kavalactones [19]. Bioactive properties include antioxidant, anti-inflammatory, fungistatic, and antibacterial activities against *Escherichia coli*, *Bacillus subtilis*, and *Bacillus cereus* [15], with proven efficacy against human immunodeficiency virus (HIV-1) and neuraminidase enzymes [20]. Additional bioactivities focus on specific obesity-related targets [10,11,12,13,23,24] and cancer [25,26].

### 4.1. Kavalactones

There are two major kavalactones in *A. zerumbet*, dihydro-5,6-dehydrokavain (DDK) and its derivative 5,6-dehydrokawain (DK) [18,19]. According to their pyrone moieties, DDK belongs to the type D and DK to the type C, since at positions 7,8 DDK has an unsaturated linkage and DK a saturated one [19]. For DDK, the highest amounts per fresh weight were detected in leaves, followed by rhizomes, and stems, whereas the reversed constellation was found for DK with the highest contents in rhizomes and lowest ones in leaves (Figure 1). Overall, the plant contains much more of the mother phytochemical DDK than its derivative DK, and the biological activities of DDK are much greater than those of DK [19]. Consequently, and under usual conditions, bioactivities of kavalactones in *A. zerumbet* are mostly related to DDK rather than to DK, but variability in kavalactone amounts is known among leaves, stems, and rhizomes, depending on location of growing and time of harvest during the year [19]. Recently, with methytriacetolactone, another kavalactone was found in leaves of *A. zerumbet* (Table 2) [27]. However, this compound was detectable only after treatment by UV (ultraviolet)-C light and is likely a derivative of either DDK or DK on degradation due to exposure to UV-C light, because it was not present in the plant prior to treatment. 

### 4.2. Non-Kavalactones

Many non-kavalactones have been identified in plant parts of *A. zerumbet* and are listed with their respective references in Table 2. Leaves of this ginger variety are rich in essential oils, mostly monoterpenes and terpenes [27]. Other plant parts such as stems, rhizomes, and flowers also contain essential oils but in much smaller amounts [27]. Additional phytochemicals are phenols, fatty acids, flavonoids, phenolic acids, labdadienes, zerumins, rutin, catechins, chalcones, and steroids (Table 2), distributed in all plant parts. In addition to kavalactones, essential oils and flavonoids have been shown to play a principal role in the biological activities of *A. zerumbet* [19,27].

Among the identified phytochemicals presented in Figure 2 and Table 2, and on a quantitative basis, DDK accounted for most of the ingredients (80–410 mg g^−^^1^ fresh weight), as compared to essential oils (<150 mg g^−^^1^), phenols (108 mg g^−^^1^), and DK (10–100 mg g^−^^1^). Negligible quantities were described for phenolic acids (<1 mg g^−^^1^) and fatty acids (62 µg g^−^^1^). Other compounds were found in much lower amounts as compared to phenolics acids and fatty acids; these other compounds were assessed by percentages of total peaks determined by gas chromatography-mass spectrometry (GC-MS), or remained unquantifiable due to missing published data (Table 2).

### 4.3. Antioxidative Phytochemicals

Many phytochemicals identified in different parts *of A. zerumbet* provide scavenging activities for free radicals and reactive oxygen species (ROS) to variable degrees (Table 2). Most important were essential oils, preferentially found in the leaves [18,19,20,24,27,30,31,32,33,34,35,36,37,38,39,40,41,42,43,44,45,46]. Less important were other compounds such as phenolic acids and flavonoids [15,18,19,27,28,30,50]. By comparison, DK and DDK failed to exert strong and specific radical scavenging activity, but this was mostly compensated by their high amounts found in every plant part [51]. Finally, the antioxidant activity of other compounds such as rutin, zerumins, quercetin, and labdadience (Table 2) was much lower as compared to essential oils, flavonoids, phenolic acids, and kavalactones. Consequently, whenever the positive antioxidative capacities of *Alpinia zerumbet* are evaluated, the percentage and total activity of each plant part must be considered. Uncertainty prevails presently with respect to which of the various phytochemicals are responsible for the Okinawa longevity or are at least partially involved.

## 5. Longevity-Specific Bioactivities of *Alpinia Zerumbet*

For *Alpinia zerumbet*, many experimental and rarely human bioactivities have been reported under in vivo and in vitro conditions. With a focus on the aspects of longevity in Okinawa, specific plant activities may be of relevance and will be discussed in detail below. Of potential clinical interest are properties such as anti-obesity, anti-lipocytes, anti-pancreatic lipase, anti-dyslipidemia, anti-atherosclerosis (including anti-low density lipoprotein (LDL) oxidation, anti-15-lipoxygease, anti-tyrosinase), anti-diabetes, anti-hypertension, and anti-tumour activities. 

### 5.1. Animal Longevity

Lifespan and in particular longevity are important parameters in clinical medicine, influenced by various exogenous and endogenous factors. With respect to these factors, animal models for studying lifespan or longevity have become popular, in view of the long period required for robust results of longevity studies in humans. One animal (nematode) model using the free-living, transparent round worm (*Caenorhabditis elegans*) was applied to study the effect of water-based extracts of *A. zerumbet* leaves on lifespan [20]. Fresh leaves were used, suggesting the *A. zerumbet* under investigation was of local Okinawan origin. In this trial, this plant significantly extended the lifespan of *C. elegans* by 22.6%. The extension was attributed to the plant’s free radical scavenging effects and its upregulation of stress-resistant gene proteins, including superoxide dismutase-3 (SOD-3) and the heat-shock protein (HSP-16.2). Genes such as Sirtuin-1 and Sirtuin-2 are increasingly under discussion for their role of determining experimental lifespan, but robust data on *A. zerumbet* are lacking.

Of note, the water fraction of the *A. zerumbet* leaves contained considerable amounts of total polyphenols, an important fact with respect to their use as teas, spices, or vegetables in the Okinawan cuisine. Overall, these animal studies show many beneficial properties of *A. zerumbet*, which suggests its potential use as a promising ingredient of a dietary supplement or as an efficient herbal drug under regulatory surveillance.

### 5.2. Anti-Obesity

Obesity is a complex and complicated metabolic disease of increased body weight due to excessive fat accumulation, caused by an imbalance between caloric uptake and energy consumption, favouring a positive energy balance [52,53,54,55]. Calculated as weight in kilograms divided by the square of height in meters, the body mass index (BMI) is a good diagnostic marker to differentiate obesity from overweight [52]. Whereas obesity is defined as BMI ≥ 30 kg m^−2^, overweight is considered as a BMI between 25 and 30 kg m^−2^. Various risk factors are closely associated with obesity, including early death, metabolic syndrome, type 2 diabetes mellitus, insulin resistance, dyslipidemia, atherosclerosis, hypertension, cardiovascular disease, and non-alcoholic fatty liver disease with potential progression to liver cirrhosis, hepatocellular carcinoma, and end-stage liver disease or acute liver failure requiring liver transplantation [52,53].

Obesity is a major global health and economic burden with an increasing tendency at an alarming rate in many countries [53]. This is a problem not shared by the Okinawa population [56]. Considering the pathogenesis of obesity and its associated diseases at the molecular level, oxidative stress with generation of toxic free radicals and reactive oxygen species (ROS), appears to play a critical role [55]. Therefore, using plants such as shell ginger with its high radical scavenging potency to counteract the injurious effects of free radicals is likely part of the efficient prevention of complications related to obesity in individuals of Okinawa to gain longevity. To achieve these positive goals, various mechanistic approaches are under consideration, as listed and discussed below.

#### 5.2.1. Anti-Lipocytes

Of interest, phytochemicals from *A. zerumbet* inhibit ROS production directly in fat cells, also called adipocytes or lipocytes [11]. Indeed, at the level of these cells, *A. zerumbet* has striking experimental anti-obesity effects elicited by DDK and DK, as well as hispidin, a derivative of DDK. This derivative can be obtained by hydrolysis in stomach acid and subsequent hepatic metabolism by an isoenzyme of cytochrome P450 (CYP), the microsomal CYP2C9 [10]. All three phytochemicals increased lipolysis when incubated with differentiated 3T3-L1 adipocytes, using glycerol release as parameter. Compared to controls, hispidin, DDK, and DK significantly increased glycerol release by 276.4%, 225.1%, and 137.1%, respectively (Table 3). All these compounds also reduced intracellular triglycerides in a dose-dependent manner. Therefore, *A. zerumbet* with its ingredients and hispidin may well constitute a new preventive or therapeutic principle for anti-obesity with lipocytes as the primary organ target, in addition to the role of liver or intestinal cells. Clearly, decreased adipocytic lipogenesis is the result of radical scavenging bioactivities from *A. zerumbet*. 

#### 5.2.2. Anti-Pancreatic Lipase

Another clinical approach to preventing or treating obesity is through retarding the intestinal absorption of dietary fat by inhibition of pancreatic lipase activity [10]. Produced in the pancreas and delivered as pancreatic juice into the duodenum, this lipolytic enzyme hydrolyses dietary triglycerides to mostly diglycerides and free fatty acids, which are readily absorbed by the epithelial cells of the small intestine. Experimental studies have shown that hispidin, DDK, and DK isolated from *A. zerumbet* strikingly reduce porcine pancreatic lipase activity likely by a process not directly involving radicals, whereby DDK and hispidin appear to be the most potent inhibitors. In other studies that compared different plant parts, extracts from seeds showed the highest inhibitory effects on pancreatic lipase activity, with response doses of 5.0–27.8 µg L^−1^ (Table 3) [12]. This leads to the conclusion that phytochemicals of *A. zerumbet* could be beneficial not only in lipocytes but also in the context of retarded intestinal fat absorption. 

#### 5.2.3. Anti-Dyslipidaemia

Typical dyslipidaemia in human obesity consists of increased triglycerides and free fatty acids, high density lipoprotein (HDL) dysfunction, decreased HDL-cholesterol, and normal or slightly increased low density lipoprotein in LDL-cholesterol [53]. Several potential interventions are under discussion to treat obesity-related dyslipidaemia in order to prevent the atherogenic lipid burden [53,57]. In experimental studies, seeds of *A. zerumbet* potentially elevate HDL-cholesterol levels, likely due to their high contents of rutin, quercetin, and polyphenols [23]. It is unclear whether other plant parts of *A. zerumbet* can ameliorate this special form of dyslipidaemia caused by obesity.

#### 5.2.4. Anti-Atherosclerosis

Atherosclerosis is one of the most serious complications associated with obesity [53,57], triggered by ROS and additional factors [58] including enzymes such as tyrosinase and 15-lipoxygenase, a mediator of LDL oxidation [12]. With respect to these key enzymes, all plant parts of *A. zerumbet* showed inhibitory effects to a variable extent, but seeds were the most powerful inhibitors of tyrosinase and 15-lipoxygenase, and LDL oxidation with response doses of 2.30–312.5 µg L^−1^, 1.26–1866.8 µg L^−1^ and 15.4–515.5 µg L^−1^, varying among rhizomes, stems, flowers, leaves, seeds, and pericarps, respectively (Table 3). It seems that shell ginger has powerful anti-atherogenic properties, which could help prevent human cardiovascular disease and stroke.

#### 5.2.5. Anti-Diabetes

Commonly observed in patients with obesity, diabetes mellitus has a major impact on the clinical outcome of the disease complex [52,53]. Advanced glycation end products are important risk factors of diabetes mellitus and their formation can be inhibited by phytochemicals derived from *A. zerumbet* [13]. The most potent inhibitor was labdadiene isolated from the rhizome, as compared to DK and DDK. Therefore, with labdadiene as an antiglycation agent, glycation-triggered complications of diabetes mellitus could be prevented.

#### 5.2.6. Anti-Hypertension

Hypertension as part of the obesity disease complex contributes heavily to morbidity and lethality [52,53]. In studies using an animal model of hypertension, hypotensive responses have been observed following treatment with essential oils of *Alpinia zerumbet* and its main constituent, terpinen-4-ol [41]. Decreases of blood pressure were dose-dependent and related mainly to vascular smooth muscle relaxation in this hypertensive model. The antihypertensive and vasorelaxant effects of *A. zerumbet* have been confirmed in a recent experimental study using a methanolic fraction of the essential oil [24]. This study also mentioned that the leaves of *Alpinia zerumbet* are used in folk medicine in Brazil to treat hypertension. Transferring these experimental data to obese humans, *A. zerumbet* could well attenuate hypertension in patients with obesity, in addition to marketed, highly effective synthetic drugs for hypertension. 

### 5.3. Anti-Tumor

Epidemiological data have shown an association between obesity defined by increased BMI and cancer across populations worldwide [55]. In men, increased risks were observed for rectal and prostate cancers, and in women for gastrointestinal, endometrial, and postmenopausal breast cancers. Ample evidence exists that cancer susceptibility in obesity is mediated in part by oxidative stress through generation of ROS. Described as strong antioxidants, most phytochemicals of *A. zerumbet* are capable of scavenging these free radicals [14,16,17,27]. This results in aqueous extracts, which commonly provide stronger antioxidant activities than the ethanol extract (Table 3) [12]. This supports the experimental studies on human tumour cell lines showing anticancer activities of *A. zerumbet* and its isolated compounds [25,26]. Among compounds isolated from *A. zerumbet*, several showed anticancer activities, reflected for example in the IC_50_ values for labdadiene: 52.1 µM; (2,5-bis(1*E*,3*E*,5*E*)-6-methoxyhexa-1,3,5-trien-1-yl)-2,5-dihydrofuran (MTD): 58.6 µM; (*E*)-2,2,3,3-tetramthyl-8-methylene-7-(oct-6-en-1-yl) octahydro-1*H*-quinolizine (TMOQ): 49.3 µM; and kaempferol-3-O-β-D-glucuronide (KOG): 12.9 µM on p21-activated kinase 1 (PAK1) (Table 3). More specifically, hydroalcoholic extract from *Alpinia zerumbet* leaves inhibited cellular proliferation only at high concentrations, while the dichloromethane extract showed a moderate antiproliferative effect against human leukaemia and lung tumour cells. Among the isolated phytochemicals, DK showed a potent cytostatic effect against glioblastoma cells and a moderate effect on all other tumour cell lines [25]. However, it is premature to introduce presently *Alpinia zerumbet* or one of its phytochemicals in clinical oncology.

## 6. Okinawa Life-Longevity

Human life is limited as lifespan depends on many variables leading to death. Among the most relevant causes of both death and reduced life span are various diseases. The top cause of deaths worldwide in 2015 was ischaemic heart disease, followed by stroke, lower respiratory infections, chronic obstructive pulmonary disease, lung cancer, diabetes mellitus, Alzheimer’s disease, diarrhoea-related diseases, tuberculosis, and road injury, according to the data of Statistica published in 2015 [30]. With respect to the United States, the leading causes of death in 2014 were heart disease, followed by cancer, chronic lower respiratory diseases, accidents, stroke, Alzheimer’s disease, diabetes mellitus, influenza and pneumonia, renal diseases, and intentional self-harm, as published by the US National Center for Health Statistics, 2016 [47]. Similar data were obtained in Europe for people over 65 by Eurostat, 2016 [48]. However, the top cause of death in Japan is cerebrovascular disease, followed by ischaemic heart disease, lower respiratory infection, lung cancer, stomach cancer, colorectal cancer, chronic kidney disease, liver cancer, and chronic obstructive pulmonary disease, as communicated by the Japan Institute of Health, 2015 [49]. 

A recent report has now identified cardiovascular disease as the top cause of death for both sexes, and neoplasms as the second leading cause of death in Japan nationally, with major variations among the prefectures [59]. Comparing Japan overall with Okinawa, the mean mortality rates from ischaemic heart disease, Alzheimer’s disease, breast cancer, and oesophageal cancer were not remarkably different. However, for Okinawa, four death categories were significantly lower, including ischaemic stroke, stomach cancer, pancreatic cancer, and liver cancer due to hepatitis C (Table 4).

In 2012, the leading cause of death in Okinawa was cancer (207.9 per 100,000) followed by heart disease (111.8), pneumonia (65.5), brain vessel disease (59.5), accident (19.2), and suicide (20.3), with increased figures since 1995 especially for cancer and heart disease as provided by hadenablog [60]. Of interest for the Okinawa prefecture, life expectancy at birth in 2015 increased by 1% for women and by 1.5% for men, in accordance with identical figures for national Japan [59]. 

A comparison of age-standardized disability-adjusted life-years (DALY) rates between Japan’s national mean and Okinawa for the global burden of diseases, injuries, and risk factors (GBD) is shown for most diseases in detail (Table 5). There were variations in many major causes of death such as ischaemic heart disease when comparing the means of Okinawa and national Japan. Of them, rates of iron-deficiency anaemia, Alzheimer’s disease, stomach cancer, diabetes, and ischaemic stroke were markedly lower in Okinawa, while other factors were indistinguishable (Table 5).

While many diseases emerge because of risk factors and inevitably lead to premature death and reduced life span, in other situations, when known risk factors are determined for an individual and can be eliminated, life span will likely be expanded. Of course, with respect to risk factors such as genetic susceptibility, people have to live with their genetics throughout their lives. The Okinawa Centenarian Study of 1976 provided additional information [61] and presented evidence-based details after examining over 900 Okinawa centenarians and numerous other elderly aged in their seventies, eighties, and nineties. These data support the view that genetic factors and the traditional Okinawan lifestyle are the most important factors contributing to longevity in Okinawa Islands.

## 7. Genetics and Traditional Lifestyle in Okinawa

### 7.1. Genetic Background

Early studies in Okinawa provided clear evidence for a genetic background of longevity in Okinawa [9]. For instance, the frequencies of 80 human leucocyte antigen (HLA) antigen phenotypes in 82 centenarians and 20 nonagenarians in Okinawa were compared with those in other healthy adults in various age brackets. Subjects aged over 90 had an extremely low frequency of HLA-DRw9 and an increased frequency of DR1. The DRw9 antigen, which occurs with a relatively high frequency in Japanese and a very low frequency in Caucasians, whilst the DR1 is associated with seronegative, rheumatoid arthritis, penicillamine-induced myasthenia, and schizophrenia. In this age group, the relative risk of HLA-DRw9 was 5.2 and for HLA-DR1, this value was 13.3. Since a high frequency of DRw9 and a low frequency of DR1 are associated with autoimmune or immune deficiency diseases, the genetic protection against these disorders may contribute to longevity [9]. In a subsequent study, HLA class II alleles of Okinawan centenarians were analysed in detail by using the polymerase chain reaction-restriction fragment-length polymorphism (PCR-RFLP) method to clarify primary genetic factors in the major histocompatibility complex (MHC) region associated with human Okinawan longevity [62]. DRB1 (associated with an increased incidence of rheumatoid arthritis) × 1401, DQB1 (associated with an increased risk of developing type 1 diabetes) × 0503, DQA1 (provides instruction for making a protein that plays a critical role in the immune system) × 0101 = 0104 and DQA1 × 05 were significantly increased in the centenarians. The significant increase of HLA-DQB 1 × 0503 and/or DQA 1 × 0101 = 0104 in the centenarians can be explained by a linkage disequilibrium with DRB 1*1401, or vice versa. Further, the tendency was observed to increase with respect to DRB1 × 0101 and DRB1 × 1201. These data suggest that several alleles of the HLA-DRB1 and/or HLA-DQ (cells can serve as antigen presenting cells in the small intestinal mucosa) are involved in human longevity [62].

### 7.2. Physical and Social Activities

Most Okinawan centenarians enjoy physical exercise, are active as walkers, and grow or once grew gardens as a form of daily physical activity. Okinawan households rarely have furniture for sitting, forcing residents to take their meals and beverages by sitting on the floor using tatami mats. Sitting down to and standing up from the floor at several occasions during the day is a crucial part of the daily physical exercise of older individuals, providing and improving body strength and balance. This also facilitates protection from falls leading to deleterious bone fractures. The role of physical activity in the healthy aging of Okinawans has been emphasized, as they keep physically active in a natural way, for instance by farming or karate. Particular exercise interventions are under study [61]. Lifestyle determinants of Okinawan women include regular exercise in the form of dance, soft historical martial arts, walking, and gardening. As such, elderly Okinawans have complete independence in terms of physical activity [56]. The levels of physical activity of male centenarians range between the categories of being completely independent and being independent but slow. In female centenarian participants, their physical activities are independent but slow or independent with difficulty [56]. Although studies on physical activities in Okinawa are limited [56,61], additional results from a large-scale population-based cohort study in Japan, the Japan Public Health Center (JPHC) Prospective Study, indicate that increased daily total physical activity is associated with significantly decreased risk of all-cause mortality in both sexes [63], conditions likely applicable also to Japanese Okinawans.

Although not quantifiable, longevity in Okinawa is facilitated likely by the local tradition of moai, established as secure social networks. These safety nets lend emotional support in times of need and give their members emotional and health security. Such psychological and social aspects are considered of utmost importance [61].

### 7.3. Caloric and Dietary Restriction

Caloric restriction is viewed as a key element of the advantage in longevity in Okinawan generations born before World War II [7,64]. This has to be differentiated from dietary restriction that may cause undernutrition or malnutrition, associated with dietary deficiencies, health problems, and reduced life span [65], conditions not observed among elderly Okinawans [56]. Indeed, this population is of a short stature, while their body weight and body mass index (BMI) values are not low, especially in elderly women, who seek to reduce their food intake for reasons of weight control [56]. As expected with advancing age, the energy intake of female centenarians is low. Among this cohort, the activities of daily life (ADL) are positively related to energy, suggesting that the low energy intake of the female centenarians was mainly due to their low levels of ADL. In the centenarian cohort as compared to the elderly, virtually all anthropometric, haematological, and biochemical variables are lower with statistical significance or near the lower reference limit, particularly in females [56]. The conclusion was reached that the nutritional status assessed by ADL and various parameters was poor in the centenarians but it was maintained (except for height) in the elderly Okinawans, and diet was not the major factor of their problems of reduced nutritional status.

If not accompanied by nutritional deficiencies, the caloric restriction implicated in Okinawan longevity is reproducible in animal models for delaying morbidity and extending average and maximum lifespan. However, the effects in humans require specific attention [64], since the anti-aging effect appears robust until the restriction reaches about half the typical calorie intake of the studied animal models [6,66]. In one human epidemiology study of Japanese-American men on calorie intake, there was a trend toward lower mortality of participants who consumed 15% less calories than the group mean, whereas an increased mortality was observed in individuals, whose caloric intake was below 50% of the group mean [66]. It seems that moderate rather than striking calorie restriction is the preferred approach for longevity.

In addition, new concepts focus on compounds including sirtuins that mimic the biochemical and functional effects of caloric restriction, as detailed in several reports presenting variable proposals [6,67]. For instance, these caloric restriction mimetics (CRMs) are thought to stimulate autophagy by favouring the deacetylation of cellular proteins in common body cells. Plausible on theoretical grounds, such deacetylation processes can be achieved by three approaches: depletion of acetyl coenzyme A, inhibition of acetyl transferases, or stimulation of deacetylase activities [67]. In our opinion, this definition of caloric restriction mimetics should be expanded specifically also to lipocytes as an additional cell type target, which would allow inclusion of lipolytic compounds that remove calories by another kind of autophagy process, namely by lysis of fat (triglycerides) out of these lipocytes. Conceptually, we suggest therefore that such a dual autophagy model better explains cellular targets involving calorie restriction mimetics, but this new hypothesis requires confirmation by further investigations. Combined with some true caloric restriction in the usual way, this additional dual autophagy process of caloric restriction mimetics should provide an optimal approach for Okinawan individuals, who use the traditional Okinawa diet and attempt not to gain weight. There is also the note that both the usual caloric restriction and the traditional Okinawan functional foods with CRM properties likely will contribute to improving health and increasing the lifespan [6].

Presumably, active but not yet identified phytochemicals of shell ginger function act as caloric restriction mimetics in the dual autophagy process. Expanding these considerations, we also propose that this dual autophagy process renders some kind of empty calories that do not count in the context of energy supply in the bodies of individuals consuming *Alpinia zerumbet*.

### 7.4. Westernized Lifestyle

As compared to other prefectures in Japan, the life expectancy advantage was previously high in Okinawa for all age groups, but it has declined since 1960 and is now limited to Okinawans of older ages due to a residual effect related to use of the traditional Okinawa diet [3]. In fact, younger Okinawans have made the switch from the traditional Okinawa cuisine to the Western food and living style. These changes in food consumption resulting in lower life expectancy clearly support the relevance of the traditional Okinawa diet for the longevity of its consumers.

## 8. Traditional Okinawa Cuisine

The traditional diet in Okinawa is low in calories but nutritionally dense due to the high preference of vegetables and fruits [8,68]. Consumption of meat, refined grains, saturated fat, sugar, salt, and full fat dairy products is limited [8]. Lacking refrigerators in earlier times and in face of the tropical climate in the Okinawa Islands, fish was difficult to preserve and was rarely consumed. Traditional Okinawa food is spicy and has a favourable appearance [69]. 

In around 1950, total daily caloric intake in Okinawans was 1785 kcal [68]. With 849 g corresponding to 69% of total calories, sweet potatoes provided most of the calories rather than rice at 154 g (12%). Contribution to the total calories was low for other potatoes (<1%) wheat, barley, and other grains (7%), nuts and seeds (<1%), sugars (<1%), oils (2%), fish (1%), meat including poultry (<1%), eggs (<1%), dairy (<1%), seaweeds (<1%), other vegetables (3%), and fruits (<1%). These data provide some details on the food composition and the caloric contribution in around 1950 in individuals of Okinawa, which differed substantially from those of mainland Japan [68]. 

Known in local dialect as *getto* in Japan and as *sannin* in Okinawa, the aromatic shell ginger enjoys high local popularity in Okinawa Islands and in the traditional Okinawa cuisine. Throughout the year, the evergreen *A. zerumbet* is abundantly and freely available from the large landscape, an important characteristic in terms of its use in larger amounts considering the overall low-income situation and associated poverty of the population in Okinawa Islands. In smaller amounts it is also taken from nearby own gardens, or local markets. Prepared from freshly harvested leaves, its use as beverage including tea is highly appreciated. Fresh leaves of shell ginger are used also for wrapping Mochi, a traditional rice cake in Okinawa [17]. Apart from tea and other beverages, shell ginger plants are a common feature of the traditional Okinawa cuisine for centuries, and are used as vegetables and spices (Table 6).

Inclusion of shell ginger for flavouring food or dressing salads instead of salt will help classify this plant as a beneficial and preventive food additive; salt is often used in excess in Western countries with deleterious health effects such as hypertension, a contributing or sole cause of many vascular diseases leading to premature death. 

Of note, the traditional approach to eating in Okinawa includes the consumption of food until reaching not full satiety but a level around 20% below complete satiety. These conditions are likely difficult to achieve in most populations outside of Okinawa and Japan, due to mental barriers. 

## 9. Challenges and Perspectives

### 9.1. Current Issues

Shell ginger, as described for culinary use in connection with food or beverages, is better classified as *A. zerumbet* for botanical purposes to clearly identify the plant, differentiating it from common ginger in the English language, which is the botanical *Zingiber officinale* used as ginger for ginger ice cream, ginger bread, ginger beverages, ginger beer, or ginger ale in many countries. Shell ginger is an exceptional multi-targeted plant for human use, considering various organs, diseases, and mechanistic actions. As it presently stands, the plant’s potential for human life is likely underestimated. The large number of highly qualified agricultural, analytical, chemical, and epidemiological studies on *A. zerumbet* and longevity in Okinawa is amazing and qualifies this topic as an important one. Many positive effects have been described for *A. zerumbet*, using experimental models that may or may not be transferrable to human conditions. Under these aspects and to err on the side of caution, it would be premature to classify *Alpinia zerumbet* as a hidden champion of preventive or therapeutic measures, although the chances are excellent, if additional compelling evidence is provided, that shell ginger is indeed a major contributor towards longevity in Okinawa. 

This present paper will help in the development of herbal drugs with *A. zerumbet* as ingredient, marketed under strict regulatory guidance. Since this plant has a long history of safe use as traditional medicine, timely regulatory approval should be received, provided clinical studies confirm its efficacy and lack of adverse effects. Less desirable is the manufacture of *A. zerumbet* in herbal dietary supplements with a much lower level of regulatory marketing restriction, insufficient to ensure consumer safety. An internet search specified a single marketed herbal dietary supplement with *A. zerumbet* as ingredient manufactured from its leaves, with a proposed daily usage of two capsules containing 250 mg each. However, additional data in publications of peer reviewed journals are not available, especially regarding efficacy, adverse effects, and contraindications; its use is therefore limited and presently not encouraged. 

The standard book of Barnes et al. published in 2007 on herbal medicines broadly discusses the family species *Zingiber officinale* Rocoe (Zingiberaceae) under the heading of ginger, representing edible plants similar to shell ginger (*A. zerumbet*), which is not explicitly mentioned [70]. As outlined, ginger is listed by the Council of Europe as a natural source of food flavouring with the category N2, which indicates that ginger can be added to foodstuffs in small quantities, with a possible limitation of a yet unspecified active principle in the final product. Used widely in food as spice, ginger has been listed as generally recognized as safe (GRAS), but some warnings and contraindications have also been added [70]. Whether these limitations apply to shell ginger has not yet been determined. However, good safety news with respect to the overall Okinawa diet can be derived from serum laboratory data of liver tests with normal values of alanine aminotransferase (ALT) and aspartate aminotransferase (AST) from supercentenarians in Okinawa [71]. Nevertheless, the efficacy and safety issues of shell ginger, and its relation to longevity in Okinawa are still a puzzle with pieces that have to be put together by additional research.

### 9.2. Future Research

The key question will be whether shell ginger can be introduced as drug to aid body weight loss or prevent body weight gain. For these indications and outside the focus of the present report, other herbs such as green tea (*Camelia sinensis* (L.) Kuntze) extracts or the chemical drug orlistat are on the market but there are conflicts with respect to potential hepatotoxicity. Also not specifically considered are chemicals and plants such as grape resveratrol, polyphenols from blueberries, phenolic antioxidants from spinach, epicatechin from green tea, and ginkgo (*Ginkgo biloba* L.), all with life-expanding effects on *C. elegans* [20].

With respect to *A. zerumbet* and longevity in Okinawa, the pioneering work at Ryukyus University of the Okinawa Islands has provided many interesting details. Future animal studies should now focus on the identification of those phytochemical(s) responsible for lifespan extension. For this research, suspected phytochemicals of *Alpinia zerumbet* should be isolated for studies using the *C. elegans* model. Experiments are encouraged for comparing lifespan extension by various plants of the Okinawa cuisine. However, since the potential efficacy is based likely on the joint actions of all phytochemicals of *Alpinia zerumbet*, the use of a single promising chemical in humans can be problematic. Likewise, efficacy of *Alpinia zerumbet* will probably be improved if used as an adjunct to the Okinawa diet or a food with similar components.

It will be the most important goal for future clinical and epidemiological investigations or new reviews to establish the exact ideal intake of shell ginger and eventually its bioactive compounds. Indeed, there is still some uncertainty from the published reports about the quantity of shell ginger (including plant parts) that the centenarians in Okinawa have actually used over their lifetimes. This question can easily be addressed by retrospective assessment of their food history with the help of expert dietitians and a special questionnaire. On this occasion, other plants with their special parts used by these individuals should also be identified and quantified. Answering all these questions will allow for recommendations that are more specific on how best to use these plants.

Clinical data are missing on to what extent shell ginger and the other plants as part of the Okinawa cuisine have actually caused a reduction in body weight. However, this would require a strict protocol designed as a metabolic ward study as in a manner published previously [72,73]. Short term efficacy is best tested using as an endpoint goal the reduction of body weight as a surrogate marker since the treatment effect on life span extension itself is impracticable in the context of initial studies. Proof of treatment efficacy requires a standard approach, using the principles of randomized clinical trials (RCTs). Consensus exists among clinicians, scientists, and manufacturers that placebo-controlled, randomized, double-blind clinical trials are the gold standard for obtaining valid results of efficiency prior to treatment initiation using herbal products [74,75]. In particular, both evidence-based medicine (EBM) and the Cochrane collaboration efforts resulted in guidelines for planning and reporting of RCTs [76]. The medical community has adopted quality criteria as per the consolidated standards of reporting trials (CONSORT) criteria. Only such metabolic ward studies combined with RCTs will provide clear-cut answers for potential consumers of shell ginger. Using this plant as a herbal drug or herbal dietary supplement may be recommendable if a there is positive benefit in relation to the risk profile. Many nutraceuticals and herbal products are highly appreciated in developed countries and are often used despite a lack of established efficacy and some suspected but not necessarily proven safety concerns [76,77,78,79,80,81,82], and the question has been raised as to whether there is good science behind the hype [74].

## 10. New Encouraging Conceptual Approaches

In terms of epidemiological and clinical studies, abundant results have been published on longevity in the Okinawa Islands, which significantly contributed to our understanding of this unique health condition and the role of obesity as a risk factor for premature death. As expected, most of the early relevant studies showed limited understanding and access to molecular aspects. 

Focusing more on molecular considerations, new encouraging concepts have become obvious. This is particularly true with respect to obesity, which can now be classified as a molecular disease, as pathogenetic mechanisms based on the generation of free radicals and ROS apply to both abdominal and peripheral obesity, and to a large number of associated diseases. Free radicals and ROS can be neutralized by the phytochemicals of many plants, including those growing in the Okinawa Islands. 

**Figure 3:**
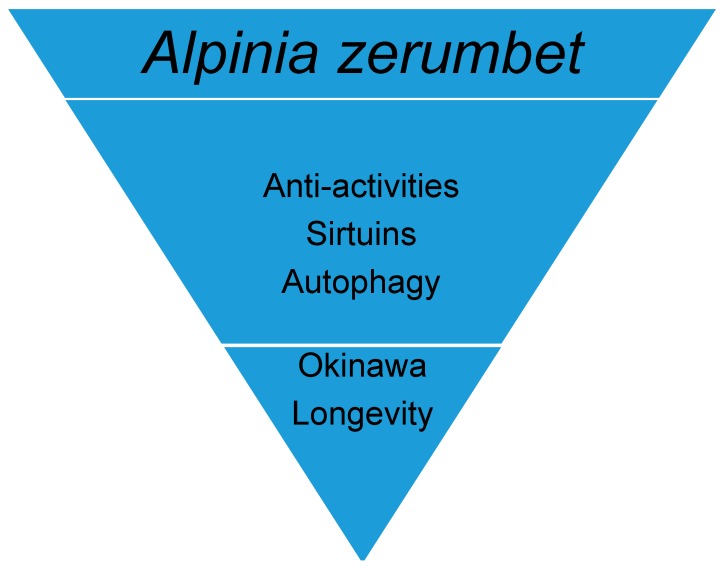
Various steps at the cellular and chemical level are presented in condensed form, leading from *Alpinia zerumbet* to longevity in Okinawa as discussed in more detail in the text.

*Alpinia zerumbet*, also called shell ginger, is a highly appreciated plant that is very popular in Okinawa, and used as traditional herbal medicine or as part of the Okinawa cuisine that is commonly considered as cause for longevity in Okinawa. Its abundant antioxidant phytochemicals can attack many obesity-related targets, and these anti-activities thus provide longevity and contribute to a healthy organism (Figure 3). 

The concept of calorie restriction mimetics (CRMs) is certainly fascinating, shown alone by the high number of publications on this topic since 1998, the year the term was coined by Lane et al. [83]. Indeed, an internet search on this term in its expanded form provided around 44,400 hits, with some of the retrieved publications addressing questions of transferability of experimental data to humans and their diseases. Among others, in scientific focus are also sirtuins [6,67,84,85,86,87] and adenosine monophosphate-activated kinase (AMPK) [84] with their potential beneficiary role in longevity [84,86,87] and the signalling network of caloric restriction [84]. In the past, many different signalling pathways and enzymatic activities have been linked to positive effects of calorie restriction [6,67,84,86]. Most of them form an interactive network, linking the regulation of transcriptional events, autophagy, and oxidative stress protection [84]. 

There is also a need to expand studies of genomic analysis including HLA in the context of longevity. Respective data published more recently show heterogeneity and inconsistencies among individuals with longevity, providing substantial genetic differences varying from country to country [88,89,90]. Indeed, partially contradictory results have been published for Okinawa [88], China [89], and the UK [90]. For instance, using genome-wide association meta-analysis of 606,059 parents’ survival in Okinawa, two regions associated with longevity (*HLA-DQA1/DRB1* and *LPA*) were newly discovered [88]. Concomitantly, previous suggestions that *APOE*, *CHRNA3/5*, *CDKN2A/B*, *SH2B3* and *FOXO3A* influence longevity were validated [88]. In China using whole-exome sequencing and sanger sequencing genotypes, a comparative population-based study with 2816 elderly people and 2819 controls revealed four new single-nucleotide polymorphisms of HLA-DQB1 (major histocompatibility complex, class II, DQ beta 1): rs41542812, rs1049107, rs1049100, and rs3891176 (*P_range_*= 0.048 − 2.811 × 10^−8^ for allele frequencies) associated with longevity in the Chinese Longevity Cohort study [89]. In the UK, two regions associated with longevity (*HLA-DQA1/DRB1* and *LPA*) were discovered, using genome-wide association meta-analysis of the survival of 606,059 parents, and previous suggestions that *APOE*, *CHRNA3/5*, *CDKN2A/B*, *SH2B3,* and *FOXO3A* influence longevity were additionally validated [90]. Most interestingly, all three studies discussed the issue of lipid metabolism [88,89,90]. In the UK, high-density lipoprotein (HDL) cholesterol levels were found to be most positively genetically correlated with lifespan [90]. In Okinawa, the effect of genetic variants on lipids metabolism was studied in long-living individuals by analysing the longevity-variants linked to blood lipids, and HLA-DQB1 rs1049107, T-carriers (*P_HDL_* = 0.006, OR: 11.277; *P_TG_* = 9.095 × 10^−7^, OR: 0.025; *P_LDL/HDL_* = 0.047, OR: 1.901), and HLA-DQB1 rs1049100, T-carriers (*P_TG_* = 1.799 × 10^−6^, OR: 0.028) were identified as being associated with lipid homeostasis in aged individuals [88]. In China, longevity and lipid homeostasis were associated with HLA-DQB1 and it was suggested that immune gene variants could act both in maintaining homeostasis and anti-aging in longevity [89]. It remains to be established whether these new or future lipid data can support or dismiss our hypothesis of dual autophagy discussed above. 

Based on convincing basic research data [6,67], a concept for *A. zerumbet* and its potential role in longevity can be formulated. *A. zerumbet* likely functions as a calorie restriction mimetic, which would in principle mimic anti-aging effects similarly to calorie restriction in experimental studies by influencing the metabolic pathways responsible for the biochemical and functional effects of calorie restriction itself. As hypothesized and supported by sirtuins and autophagy (Figure 3), CRMs lead to calories that are “empty”, and thus not suitable for effective energy supply in the bodies of individuals consuming *A. zerumbet*.

## 11. Conclusions

The Okinawa Islands in Japan are famous for their high numbers of centenarians, who consume a local delicious traditional cuisine known for its reduced caloric values and high plant content. For beverages including tea, dishes, and spices, the Okinawa population has commonly used shell ginger with the botanical name *A. zerumbet* of the ginger family (Zingiberaceae) for many centuries. Results of studies published recently have shown that this plant contains abundant bioactive phytochemicals, with striking anti-obesity, antioxidant, and anti-aging properties. In addition, animal studies classified shell ginger as a life-expanding plant and suggested that this plant, if used in humans, could expand the lifespan by 22.6%. Therefore, supportive evidence is provided for the proposal that shell ginger contributes significantly to human longevity in the population of Okinawa in Japan. 

## Figures and Tables

**Table 1:** Histogram of *Alpinia zerumbet.*

Category	Description
Preferred scientific name	*Alpinia zerumbet* (Pers.) B.L. Burtt & R.M. Sm.
Preferred common name	Shell ginger
Taxonomic tree	Domain: Eukaryota; Kingdom: Plantae; Phylum: Spermatophyta; Subphylum: Angiospermae; Class: Monocotyledonae; Order: Zingiberaceae; Genus: *Alpinia*; Species: *Alpinia zerumbet*
Other scientific names	*Alpinia speciosa* (J.C.Wendl.) K. Schum.; *Amomum nutans* (Andrews) Schult.; *Catimbium speciosum* (J.C.Wendl.) Holttum; *Costus zerumbet* Pers.; *Languas speciosa* (J.C.Wendl.) Small; *Renealmia nutans* Andrews; *Zerumbet speciosum* J.C.Wendl.
International common names	English: light galangal; pink porcelain lily; shell flower; French: *atoumau*; Chinese: *yan shan jiang*
Other names in distributed countries	Japan: *getto*; Brazil: *colonia*; Cook Islands: *kaopui*; Cuba: *boca de lobo*; Dominican Republic: *burriquito*; Haiti: *de tui maux; tous maux*; Indonesia: *galoba merah*; Myanmar: *padegaw-gyi*; the Philippines: *langkuas na pula*; Puerto Rico: *boca de dragon*; Thailand: *khaa khom*; Tonga: *teuila*; Vietnam: *Riềng ấm*
Distribution	*South, Southeast, and East Asia (from India to Japan), and southward to Oceania and Australia. Cultivated as an ornamental plant in tropics and subtropics.*
Uses	A spice, food, medicine, beverage, pharmacy, cosmetics, paper, and ornament
References	Information in this histogram is from literature and references [10,11,12,13,14,15,16,17,18,19,20,21,22].

**Table 2:** Phytochemicals in various plant parts of *Alpinia zerumbet*.

Chemical Name	Plant Parts	Quantity	References
Kavalactones Dihydro-5,6-dehydrokawain (DDK)	Leaves, stems, rhizomes, fruits	80–410 mg g^−1^	[18,27,28,29]
5,6-Dehydrokawain (DK)	Leaves, stems, rhizomes, fruits	10–100 mg g^−1^	[18,19,27,28]
Methyltriacetolactone	Leaves	0.44% **	[26]
Non-kavalactones Essential oils	Leaves, petal, roots	<150 mg g^−1^	[18,19,20,24,27,30,31,32,33,34,35,36,37,38,39,40,41,42,43,44,45,46]
(*E*)-labda-8(17)-12-diene-15-ol-16-al	Rhizomes		[47]
(*E*)-15,16-bisnorlabda-8(17)-11-diene-13-one	Rhizomes		[47]
Phenolic acids	Leaves, stems, rhizomes	<1.0 mg g^−1^	[15,18,27,29]
Phenols	Leaves	108.27 mg g^−1^	[27]
12-Labdaiene-15,16-dial (labdadiene)	Rhizomes, stems, leaves, flowers, pericarps, seeds	0.75–1.0 mg g^−1^	[12,48]
Rutin	Leaves, seeds		[23,49]
Kaempferol-3-*o*-glucuronide	Leaves		[28,49]
Quercetin	Seeds		[23]
(+) Catechin	Leaves		[49]
(−) Epicatechin	Leaves		[49]
*p*-hydroxycinnamaldehyde	Rhizomes		[21]
(i-(*p*-hydroxy-*cis*-styryl)) methane	Rhizomes		[21]
Kaempferol-3-*o*-rutinoside	Leaves		[28]
Dihydroflavokavain B	Rhizomes		[15]
Chalcones (2′,4′-Dihydroxy-6′-methoxy chalcone; 2',4'-dihydroxy-5'-methoxy chalcone)	Seeds, leaves	0.25% *	[27]
*trans*-1-(4′-Hydroxy-3′-methoxyphenyl-7-phenylhept-1-en-3-one (yakuchinone-B)	Pericarps		[22]
Labda-8(17),12-diene-15,16-dial	Rhizomes		[28]
Bisabolanes	Leaves		[28]
Steroids	Leaves, seeds	<1% *	[27]
Fatty acids	Leaves	62 µg g^−1^	[27]
Phenanthrin	Leaves	4.65% *	[27]

Symbols: * percentage of peak in comparison with total peaks determined by gas chromatography-mass spectrometry (GC-MS); ** quantified after UV (ultraviolet)-C treatment of *A. zerumbet.*

**Table 3:** Dose responses to pharmaceutical and biological activities of *Alpinia zerumbet*.

Activity	Extracts and Doses	References
Antioxidant DPPH	Aqueous (IC_50_): 10.3–165.6 µg mL^−1^ Ethanol (IC_50_): 136.6–599.0 µg mL^−1^	[13]
Antioxidant ABTS	Aqueous 73.9–163.4 µg mL^−1^ Ethanol: 137.9–293.9 µg mL^−1^	[13]
Antioxidant PMS-NADH	Aqueous: 58.6–215.1 µg mL^−1^ Ethanol: 56.1–210.5 µg mL^−1^	[13]
Total phenols	Aqueous: 47.8–187.8 mg g^−1^ GAE Ethanol: 9.6–56.3 mg g^−1^ GAE	[13]
Tyrosinase	2.30–312.5 µg L^−1^	[12]
Pancreatic lipase	5.0–27.8 µg L^−1^	[12]
15-Lipoxygenase	1.26–1866.8 µg L^−1^	[12]
LDL oxidation	15.4–515.5 µg L^−1^	[12]
Longevity	Leaf extracts: 50–100 µg mL^−1^ increased 22.6% life span of *Caenorhabditis elegans*	[20]
Anti-cancer on A549 cell line	Compounds isolated from *A. zerumbet*: IC _50_ values: labdadiene: 67.1 µM; MTD: 98.9 µM; TMOQ 90.8 µM; KOG 81.4 µM	[26]
PAK1 inhibitory	Compounds isolated from *A. zerumbet*: IC_50_ value: labdadiene IC_50_: 52.1 µM; MTD: 58.6 µM; TMOQ 49.3 µM; KOG 12.9 µM	[26]
Anti-obesity	Compounds including hispidin, DK, and DDK isolated from *A. zerumbet*: Dose of 250 µg mL^−1^. Glycerol release increased 276.4%, 225.1%, and 137.1%; cAMP promoted 81.2%, 67.0%, and 56.9%; Inhibition on lipid accumulation: 47.8%, 48.0%, and 36.8%, respectively.	[10]

ABTS: 2,2’-aino-bis(3-ethylbenzothiazoline-6-sulfonic acid) diammonium salt; cAMP: cyclic adenosine monophosphate; DK: 5,6-dehydrokawain; DDK: dihydro-5,6-dehydrokawain; DPPH: 1,1-diphenyl-2-picrylhydrazyl; GAE: gallic acid equivalent; IC: inhibitory concentration; KOG: kaempferol-3-O-β-D-glucuronide; LDL: low density lipoprotein; MTD: (2,5-bis(1*E*,3*E*,5*E*)-6-methoxyhexa-1,3,5-trien-1-yl)-2,5-dihydrofuran; NADH: nicotinamide adenine dinucleotide-reduced; PAK1 (p21-activated kinase 1); PMS: phenazine-metho-sulfate; TMOQ: (*E*)-2,2,3,3-tetramthyl-8-methylene-7-(oct-6-en-1-yl) octahydro-1*H*-quinolizine.

**Table 4:** Age-standardized rates per 100,000 of mortality by GBD for Japan overall and Okinawa in 2015 for both sexes combined [59].

	a	b	c	d	e	f	g	h
**Japan**	44.7 *	31.7 *	25.1 ^†^	18.2 ^†^	10.3 ^†^	8.3 ^†^	5.7 *	4.6 *
**Okinawa**	46.6 *	29.9 *	19.7 ^†^	12.1 ^†^	7.8 ^†^	5.9 ^†^	5.4	4.8 *

Details: a. Ischaemic heart disease; b. Alzheimer’s disease; c. Ischaemic stroke; d. Stomach cancer; e. Pancreatic cancer; f. Liver cancer due to hepatitis C; g. Breast cancer; h. Oesophageal cancer. Significance was set at *p* < 0.05. GBD: global burden of diseases, injuries, and risk factors. * Indistinguishable from national mean; ^†^ Significantly lower than national mean.

**Table 5:** Age-standardized rates per 100,000 of DALYs by GBD between Japan and Okinawa in 2015 for both sexes combined [59].

	a	b	c	d	e	f	g	h	i	j	k	l
**Japan**	813.2 *	466.8 *	438.5 *	437.9 ^†^	373.5 ^†^	341.5 ^†^	298.4 ^†^	290.0 *	280.0 *	275.0 ^†^	264.3 *	246.8 *
**Okinawa**	813.1 *	472.1 *	443.8 *	400.6 ^†^	353.4 ^†^	234.0 ^†^	213.5 ^†^	286.1 *	279.9 *	236.6 ^†^	264.4 *	251.2 *

Details: a. Lower back pain; b. Major depression; c. Migraine; d. Iron-deficiency anaemia; e. Alzheimer’s disease; f. Stomach cancer; g. Diabetes; h. Falls; i. Neck pain; j. Ischaemic stroke; k. Anxiety disorder; l. Schizophrenia. Significance was set at *p* < 0.05. DALY: disability-adjusted life-years; GBD: global burden of diseases, injuries, and risk factors. * Indistinguishable from national mean; ^†^ Significantly lower than national mean.

**Table 6:** Details of commonly used products derived from *Alpinia zerumbet* among the population in Okinawa.

*Alpinia*-derived products in Okinawa	Details of *Alpinia* preparation and use in the traditional Okinawa cuisine
Tea	One pack includes dried *Alpinia* powder from leaves, flowers, and rhizome. Few teas use only the leaves, flowers, or rhizome individually, others use mixtures. The amount of one pack is on average 2.5 g, dipped in 200–300 mL of hot water. Daily or intermittent consumption.
Mochi	Traditional sticky rice cake that is sold commonly in Okinawan markets. The rice cake is prepared with brown sugar or local purple sweet potato, covered with several fresh leaves of *Alpinia*, and steamed. When consumed, the rice cake has a strong characteristic flavour of *Alpinia* due to its essential oils, similar to the common ginger vegetable. Most Okinawans eat this cake regularly, some intermittently.
Steamed bun	Common rice powder prepared with meat or other ingredients, covered with fresh *Alpinia* leaves and steamed: not used daily but restricted to special occasions.
Fried meat or fish	The meat or fish is covered with fresh *Alpinia* leaves, steamed, and then fried before consumption on a non-regular basis.
Buckwheat	The dried powder of *Alpinia* leaves is mixed with buckwheat powder, boiled, and consumed.
Ice cream	Uses extract of *Alpinia* leaves to provide a fragrance to ice cream: it is consumed on special occasions.

Note: *Alpinia* refers specifically to *Alpinia zerumbet*. Quantification of daily *Alpinia* use among households and individuals is difficult and variable. Other products derived from *Alpinia* include soaps and cosmetics prepared from essential oils and extracts of *Alpinia*. The cosmetics are generally skin care products, although *Alpinia* is also commonly used to treat sunburn. The powder of *Alpinia* is also used as an additive for bath water.
